# Rational Prescribing in Primary care (RaPP): process evaluation of an intervention to improve prescribing of antihypertensive and cholesterol-lowering drugs

**DOI:** 10.1186/1748-5908-1-19

**Published:** 2006-08-25

**Authors:** Atle Fretheim, Kari Håvelsrud, Andrew D Oxman

**Affiliations:** 1 Norwegian Knowledge Centre for the Health Services, PB 7004 St. Olavs plass, N-0130 Oslo, Norway

## Abstract

**Background:**

A randomised trial of a multifaceted intervention for improving adherence to clinical practice guidelines for the pharmacological management of hypertension and hypercholesterolemia increased prescribing of thiazides, butdetected no impact onthe use of cardiovascular risk assessment toolsor achievement of treatment targets. We carried out a predominantly quantitative process evaluation to help explain and interpret the trial-findings.

**Methods:**

Several data-sources were used including: questionnaires completed by pharmacists immediately after educational outreach visits, semi-structured interviews with physicians subjected to the intervention, and data extracted from their electronic medical records. Multivariate regression analyses were conducted to explore the association between possible explanatory variables and the observed variation across practices for the three main outcomes.

**Results:**

The attendance rate during the educational sessions in each practice was high; few problems were reported, and the physicians were perceived as being largely supportive of the recommendations we promoted, except for some scepticism regarding the use of thiazides as first-line antihypertensive medication. Multivariate regression models could explain only a small part of the observed variation across practices and across trial-outcomes, and key factors that might explain the observed variation in adherence to the recommendations across practices were not identified.

**Conclusion:**

This study did not provide compelling explanations for the trial results. Possible reasons for this include a lack of statistical power and failure to include potential explanatory variables in our analyses, particularly organisational factors. More use of qualitative research methods in the course of the trial could have improved our understanding.

## Background

From April 2002 to December 2003, we conducted a cluster randomised-controlled trial with 146 general practices in Norway – the Rational Prescribing in Primary care (RaPP) trial [1]. We tested the effectiveness of a multifaceted intervention we had developed for improving adherence to clinical practice guidelines for the pharmacological treatment of hypertension and hypercholesterolaemia. The main recommendations we set out to implement were:

• assessment of cardiovascular risk before deciding to start antihypertensive or cholesterol-lowering treatment,

• use of thiazides as the first-line antihypertensive drug, and

• achievement of treatment goals among patients started on medication

The intervention was multifaceted and included an outreach visit conducted by one of four pharmacists recruited and trained specifically for this purpose. The pharmacist extracted information from the electronic medical records on the practice's prescribing of antihypertensives, the level of cardiac risk among patients started on treatment, and the proportion of patients that had reached recommended treatment goals. The pharmacist then met with the physicians in their practice environment and presented recommendations on the prescribing of antihypertensive and cholesterol-lowering therapy. The physicians were invited to comment on the recommendations, and data that had been extracted from their medical records were fed back to them.

During the outreach visit, the pharmacist also installed software, which triggered reminders on the computer screens when physicians were seeing patients relevant to the recommendations. The software also enabled the physicians to estimate cardiovascular risk and to print out patient information.

The results from the trial demonstrated that the intervention effectively increased the prescribing of thiazides, but no effect was demonstrated on the assessment of cardiovascular risk or on the extent to which treatment goals were achieved [1].

Prospectively, we decided to carry out a process evaluation of the implementation of the intervention. This was motivated by the belief that recording various process measures can provide insight into how the intervention was perceived and implemented in clinical practice, and that exploring this information could aid the interpretation of trial-results [2]. We had hypothesised that the impact of the intervention would be correlated to several variables, including practice specific factors such as the attitude among the physicians toward the recommendations, and process measures, such as the proportion of physicians attending the educational outreach visit. Thus, the main objective of this analysis was to identify factors that could explain variation in outcomes across practices.

## Methods

### Data collection

A logbook was kept throughout the project, and the two lead investigators (AF and KH) made notes each time there was contact with participating practices.

After each outreach visit the pharmacists completed a questionnaire addressing various aspects of the visit, such as the number of physicians attending the educational session and the pharmacist's impression of how the physicians reacted to the clinical practice guidelines (table 2). For most responses we used 5-point scales, ranging from "negative" to "positive."

**Table 2:** Pharmacists' perception of outreach visit with physicians

	1 Very negative n (%)	2 Negative n (%)	3 Neutral n (%)	4 Positive n (%)	5 Very positive n (%)	Do not know n (%)	Mean score
What was their attitude toward using the software?	0	0	3 (5)	25 (38)	38 (58)	0	4.5
What was their attitude toward printing out patient information?	0	0	28 (42)	29 (44)	7 (11)	2 (3)	3.7
How did they respond to receiving the full version of the guidelines?	0	0	12 (18)	43 (65)	11 (17)	0	4.0
How did they respond to receiving the short-version of the guidelines?	0	0	9 (14)	44 (67)	13 (20)	0	4.1
To what extent were they interested in the topic?	0	0	4 (6)	23 (35)	39 (59)	0	4.5
What was their attitude toward you?	0	0	1 (2)	47 (71)	17 (26)	1 (2)	4.2
What was their attitude to the use of cardiovascular risk assessment?	0	1 (2)	0	17 (26)	47 (71)	1 (2)	4.7
What was their attitude toward the recommendation of thiazides as first-choice drug?	1 (2)	10 (15)	24 (37)	28 (43)	2 (3)	0	3.3
What was their attitude toward the treatment goals?	0	2 (3)	8 (12)	42 (65)	10 (15)	3 (5)	4.0
How do you rate your own performance during the presentation?	0	1 (2)	7 (11)	55 (85)	1 (2)	1 (2)	3.9

All practices in the intervention-group were telephoned by one of the investigators (KH) 1–3 days after the visit, to enquire about any difficulties that had been encountered.

Within three months after the outreach visit, we conducted semi-structured telephone interviews with physicians in the intervention-practices, asking about how the intervention was perceived and their attitudes towards the recommendations we were trying to implement (table 3). The response options were "yes" or "no," or on a 3-point scale (usually "negative," "neutral," "positive"), followed up by an open question, such as "Why?". The interviews were done by one of the investigators (AF or KH) or one of the pharmacists who also conducted the outreach visits. Responses to open-ended questions were coded in categories independently by AF and KH. Disagreements were resolved by discussion. We offered a small compensation to the physicians for participating (NOK 350).

**Table 3:** Feed-back from physicians (telephone interviews)*

	Yes n (%)	No n (%)	Partly n (%)	
Are the reminders working as they should?	104 (70)	19 (13)	26 (17)	

	Negative n (%)	Neutral n (%)	Positive n (%)	Do not know n (%)

What is your attitude toward receiving reminders about cardiovascular risk assessment?	14 (9)	33 (22)	95 (64)	4 (3)
What is your attitude toward receiving reminders about treatment goals?	15 (10)	29 (20)	95 (64)	5 (3)

	Yes n (%)	No n (%)	Yes and No n (%)	

Do you usually estimate cardiovascular risk before starting antihypertensive or cholesterol-lowering therapy?	92 (62)	48 (32)	7 (5)	
Are thiazides your first choice for the treatment of uncomplicated hypertension?	52 (35)	86 (58)	9 (6)	

	Few n (%)	Some n (%)	Most n (%)	Do not know n (%)

Do you have the impression that your patients achieve recommended treatment goals?	4 (2)	34 (23)	105 (71)	6 (4)

* N = 149. Not all physicians responded to all questions.

Data on prescribing and achievement of treatment goals were extracted from the electronic medical records. The data on prescribing enabled us to identify patients that had been started on medication, and we asked physicians (by telephone) about whether they had conducted cardiovascular risk assessment first.

### Analyses

We selected potential explanatory variables for each main outcome based on our own judgement and discussion with a general practitioner. We conducted initial, univariate regression analyses to explore the association between the selected variables and the observed variation across practices. The variables that predicted the dependant variable at a statistical significance-level of p < 0.30 were included in the main analysis, which was a multivariate regression model for each main outcome. Units of analysis were the practices, and the calculations were done using the enter-command in SPSS 12.

For two outcomes (prescribing of thiazides and achievement of treatment-goals) we had measurements from before and after the intervention, and we used difference as the dependant variable. For assessment of cardiovascular risk we only had post-intervention data, which we used as the dependant variable.

## Results

Out of 388 invited practices, 146 agreed to participate by returning a signed informed consent document. In most cases, no specific reason was stated for not wanting to take part. The location and size (number of physicians) of the 73 practices randomised to the intervention group are summarised in Table 1. We managed to collect outcome-data from 70 of the 73 intervention practices. For three of the 70 practices, we were unable to complete outreach visits to, and the pharmacist questionnaire was missing for one visit. Thus, completed questionnaires by pharmacists were available for 66 outreach visits.

**Table 1:** Characteristics of practices randomised to receiving intervention

	n (%)
Location	
• Oslo-area	63 (86)
• Tromsø-area	10 (14)

Number of physicians per practice*	
• One	16 (23)
• Two	22 (31)
• Three	15 (21)
• Four	7 (10)
• Five or more	11 (15)

*Data missing from two practices

On average, 2.3 physicians per practice attended the meeting with the pharmacist (interquartile range 1 to 3), corresponding to an average attendance rate of 85% (interquartile range 67 to 100). The meetings lasted an average of 33 minutes (interquartile range: 30 to 40). Seven individual physicians declined having software for reminders installed on their computer.

The pharmacists' perceptions of physician-attitudes during the outreach visits are shown in Table 2. In general, the physicians were perceived as being agreeable to all aspects of the outreach visit, except for the recommendation that thiazides should be used as first-line medication.

The practices rarely reported problems when we telephoned them 1–3 days after the outreach visit had taken place.

### Feed-back from physicians

An estimated 195 physicians were eligible for the survey, out of which we managed to interview and complete the questionnaire for 149 (76%).

Summaries of the responses are presented in Table 3. The physicians were generally positive to receiving reminders about treatment goals and of assessing cardiovascular risk. A majority also stated that they usually assessed the cardiovascular risk and that they believed most of their patients achieved recommended treatment goals. However, eighty-six respondents (58%) reported not using thiazides as the first-choice medication. When asked why, the most common responses were fear of side-effects (19 respondents), insufficient blood-pressure lowering effect (15), and influence from pharmaceutical industry (11). Many respondents did not give a reason for not using thiazides other than old habit and tradition (6), that the drugs are considered old-fashioned (5), or simply having a preference for other drug classes (25).

The final question in the interview was about general aspects of participating in the research project: What was good? How could it have been more useful? In response to this, 58 (39%) of the physicians mentioned reminders as a useful tool. However, 21 (14%) brought up the issue that reminders interrupted them in their work. Eight respondents (5%) mentioned that the risk assessment tools were helpful when used jointly with patients.

### Regression analyses

**Figure 1:**
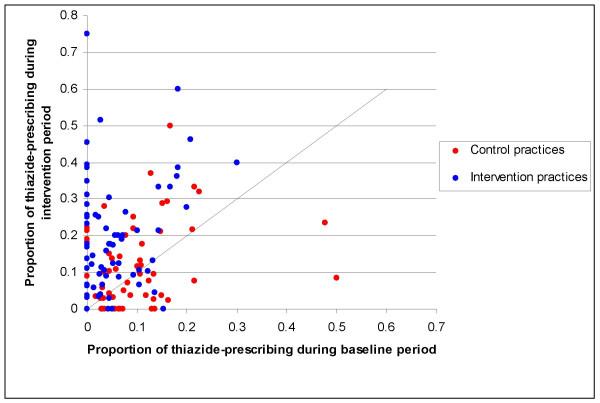
Variation in change in thiazide-prescribing among all practices in trial.

**Figure 2:**
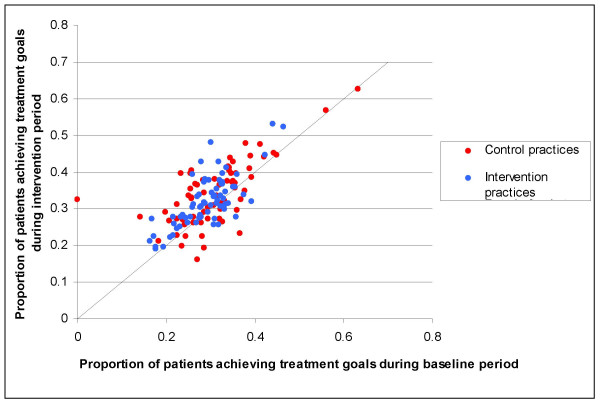
Variation in change in achievement of treatment goals among all practices in trial.

The degree of change in thiazide-prescribing varied considerably across practices (figure 1), while the change in achievement of treatment goals was more uniform, and in most cases close to zero (figure 2). There was wide variation across practices in the extent to which doctors were using cardiovascular risk assessment tools. Proportions ranged from zero to 100% (median 5%, mean 17%). We did not have baseline measurements for this outcome, thus we could not estimate whether there had been a change in performance from before to after the intervention.

The results from the initial univariate regression exercises are listed in Table 4. The resulting multivariate regression models with changes in thiazide-prescribing and achievement of treatment goals as dependant variables are found in Table 5. The multivariate regression model with rates of cardiovascular risk assessment as dependant variable is found in Table 6. The basis population for all analyses included the 66 practices for which we had completed questionnaires by pharmacists.

**Table 4:** Univariate regression analyses: Statistical significance (p-values) of explanatory variables

**Explanatory variable**	**Dependant variable**		
	**Change in rate of thiazide-prescribing**	**Change in rate of achievement of treatment goals**	**Rate of assessment of cardiovascular risk**

Geographical area (Oslo- or Tromsø-area)	0.89	0.42	0.74
Size of practice (number of doctors)	0.36	0.046	0.95
Proportion of doctors present at meeting	0.04	0.32	0.25
Pharmacist dummy-variable 1 (pharmacist A = 1, else = 0)	0.09	0.49	0.70
Pharmacist dummy-variable 2 (pharmacist B = 1, else = 0)	0.72	0.15	0.80
Pharmacist dummy-variable 3 (pharmacist C = 1, else = 0)	0.02	0.39	0.80
Length of educational meeting (minutes)	0.15	0.62	0.54
Proportion of doctors declining installation of software	0.70	0.13	0.77
Doctors' attitude toward using software*	0.83	0.62	0.67
Doctors' attitude toward printing out patient-information*	0.12	0.33	0.77
Doctors' attitude toward receiving the guidelines*	0.77	0.72	0.72
Doctors' attitude toward recommendation of cardiovascular risk-assessment*	Not applicable	Not applicable	0.22
Doctors' interest in topic*	0.55	0.92	0.16
Doctors' attitude toward pharmacist*	0.70	0.96	0.47
Doctors' attitude toward recommendation of thiazides as first-line antihypertensives*	0.32	Not applicable	Not applicable
Self-assessed performance*	0.81	0.76	0.84
Are reminders working as they should?†	0.59	0.87	0.22
Attitude toward receiving reminders about risk assessment†	Not applicable	Not applicable	0.95
Attitude toward receiving reminders about treatment goals†	Not applicable	0.002	Not applicable
Baseline rate of thiazide-prescribing	0.10	Not applicable	Not applicable

* Assessed by pharmacists after or during outreach visit, see Table 3 for coding of variable.

† Response from doctors during telephone-interviews, see Table 2 for coding of variable.

**Table 5:** Multivariate regression models: Change in thiazide-prescribing and in achievement of treatment goals

**Dependant variable: Change in rate of thiazide-prescribing**				
**Explanatory variable**	**B**	**(95 % CI)**	**Standard error of B**	**p-value**

(Constant)	0.06	(-0.21 to 0.33)	0.13	0.66
Proportion of doctors present at meeting	0.12	(-0.04 to 0.27)	0.08	0.13
Pharmacist dummy-variable 1 (pharmacist A = 1, else = 0)	-0.006	(-0.09 to 0.08)	0.04	0.89
Pharmacist dummy-variable 3 (pharmacist C = 1, else = 0)	0.05	(-0.03 to 0.13)	0.04	0.23
Length of educational meeting (minutes)	0.003	(-0.002 to 0.007)	0.002	0.21
Doctors' attitude toward printing out patient-information*	-0.03	(-0.07 to 0.02)	0.02	0.21
Baseline rate of thiazide-prescribing	-0.47	(-1.05 to 0.10)	0.29	0.11
R^2 ^= 0.21, N = 63				

**Dependant variable: Change in rate of achievement of treatment goals**				

**Explanatory variable**	**B**	**(95 % CI**	**Standard error of B**	**p-value**

(Constant)	-0.003	(-0.06 to 0.05)	0.03	0.92
Size of practice (number of doctors)	-0.006	(-0.02 to 0.003)	0.005	0.21
Pharmacist dummy-variable 2 (pharmacist B = 1, else = 0)	-0.03	(-0.07 to 0.008)	0.02	0.11
Proportion of doctors declining installation of software	0.04	(-0.02 to 0.11)	0.03	0.20
Attitude towards receiving reminders about treatment goals^†^	0.07	(0.02 to 0.12)	0.03	0.01
R^2 ^= 0.24, N = 60				

* Assessed by pharmacists after or during outreach visit, see Table 3 for coding of variable.

† Response from doctors during telephone-interviews, see Table 2 for coding of variable.

**Table 6:** Multivariate regression model: Rate of cardiovascular risk assessment

**Dependant variable: Rate of assessment of cardiovascular risk**				
**Explanatory variable**	**B**	**(95 % CI)**	**Standard error of B**	**p-value**

(Constant)	-0.12	(-0.88 to 0.64)	0.38	0.75
Proportion of doctors present at meeting	0.25	(-0.06 to 0.56)	0.16	0.11
Doctors' attitude towards recommendation of cardiovascular risk-assessment*	-0.10	(-0.22 to 0.02)	0.06	0.11
Doctors' interest in topic*	0.09	(-0.02 to 0.21)	0.06	0.10
Are reminders working as they should?^†^	0.16	(-0.07 to 0.39)	0.11	0.17
R^2 ^= 0.13, N = 61				

* Assessed by pharmacists after or during outreach visit, see Table 3 for coding of variable.

† Response from doctors during telephone-interviews, see Table 2 for coding of variable.

The models could only explain a small proportion of the observed variation in outcomes across practices (R^2 ^less than 25% for all models). There was not much overlap of explanatory variables for the different dependant variables. Only one explanatory variable came out statistically significant (p < 0.05) in the multivariate models – the association between doctors' self-reported attitude toward reminders about treatment goals, and achievement of treatment goals. If the doctors were positive, this was associated with a 7% absolute increase in achievement of treatment goals compared to doctors who were negative.

## Discussion

In general, we have found that the participating physicians had a positive attitude toward most aspects of the intervention. The attendance rate during the educational sessions in each practice was high, few problems were reported, and the physicians were perceived as being largely supportive of the recommendations we promoted, except for widespread scepticism about the use of thiazides as the first-line antihypertensive medication.

In the trial, the intervention was shown to have an impact on the rate of thiazide-prescribing, but no effects were demonstrated on the use of cardiovascular risk assessment before initiating antihypertensive or cholesterol-lowering medication, or on the degree to which patients achieved recommended treatment goals. This was surprising considering the lack of enthusiasm regarding the use of thiazides and the unanimous support of cardiovascular risk assessment and recommended treatment goals. However, doctors' attitudes had been identified as a likely reason why it could be difficult to increase the use of thiazides, and our multifaceted intervention was specifically tailored to target barriers to change, including attitudes [3].

There was a high level of agreement between the way the physicians' attitudes were perceived by the pharmacists during outreach visits, and what the physicians themselves reported during interviews. This indicates that our assessments of physician-attitudes were likely valid. However, there was a weaker relationship between how doctors perceived their own behaviour and what we found using data from medical records. For instance, we found that risk assessment had been done in 17% of cases before patients were started on medication, while 62% of the physicians claimed that they usually did this. This discrepancy may be partly due to social desirability bias: The interviews were conducted by a member of the research team, often by the same pharmacist that had visited the practice a few months earlier.

Our findings shed only limited explanatory light on the trial-results. Multivariate regression models could only explain a small part of the observed variation, and we did not identify key predictive factors for the design or implementation of a successful intervention.

A weakness of our process evaluation is the lack of more in-depth qualitative methods, e.g. in-depth interviews or focus-groups with general practitioners that could have increased our understanding of the trial-results [4]. Another weakness is that we did not have baseline data for the outcome measure for the use of risk assessment tools, which meant that we could not estimate change in performance in relation to the intervention. Whether the post-intervention rate is a valid effectiveness measure is highly questionable.

The attitudes among doctors, as perceived by the pharmacists, were rarely negative; thus our models are not necessarily applicable to practices where more negative attitudes dominate

More use of theory-based approaches has been suggested for the design of interventions to improve professional practice [5]. We applied our own OFF-theory [6] in the design of this intervention, but we did not find it to be of much use. Thus, we maintain our scepticism to theory-based approaches [6].

There are several possible reasons why we failed to find good explanations for the trial results. Many of the explanatory variables we used were based on pharmacists' impressions during outreach visits or self-reporting from physicians, and these may be inaccurate. In addition, we may simply have had too little statistical power to detect important factors. Finally, it is possible that key explanatory variables have not been included in our analysis. Some possible explanations that we have not explored include:

• Turn-over of doctors that could be expected to negatively influence the trial results;

• Impact of patient expectations;

• Organisational factors, e.g. lack of time during appointments to carry out risk assessment, or lack of systems to ensure appropriate follow-up of patients; and

• Lack of specific incentives for physicians to adhere to recommendations, e.g. compensation for extra time spent on risk assessment.

Why was the intervention more effective in terms of influencing decisions on prescribing than for the other outcomes? Firstly, we believe that selecting a drug is a fairly straightforward process that is mainly influenced by knowledge and attitudes, both of which can be addressed through an educational intervention. Secondly, assessment of cardiovascular risk may be perceived as time-consuming, and many doctors may have had a threshold for starting to use a new tool. Finally, achievement of treatment goals probably depends as much on patient behaviour as on the actions taken by doctors. The mixed effectiveness cannot be explained by baseline performance, which was low for all outcomes.

Our trial-results are relatively consistent with the findings from a systematic review of randomised trials, where outreach visits were found to be effective for changing prescribing, while the impact on other aspects of professional behaviour was more variable [7]. Similarly, the results of a systematic review of interventions to improve control of blood pressure indicated that educational interventions were "unlikely to be associated with large net reductions in blood pressure by themselves [8]. " The authors concluded that it is necessary to have "an organized system of regular follow-up and review" of hypertensive patients.

Others also have attempted to explain findings from trials of interventions to improve professional practice using process evaluations. Nazareth and colleagues studied the various processes that may lead to change in prescribing habits within the framework of the Evidence Based Out Reach trial [9]. The experiences and views of the pharmacists that conducted the outreach visits were collected using semi-structured assessment sheets and nominal group techniques. Feedback from physicians subjected to the educational outreach visits were gathered using mailed questionnaires. The authors observed a smaller effect on the uptake of the guideline in practices where the pharmacists were unable to meet all the doctors at the outreach visit, which is consistent with our own observation (table 5). The authors also noted that "Despite the positive views expressed by both the pharmacists and the GPs, we only observed a modest effect," which is comparable to what we have found.

Flottorp and colleagues also conducted a trial of a multifaceted intervention for guideline implementation in primary care, without educational outreach visits [10]. They found little or no effect from the intervention. However there was large variation among practices with regards to the degree of change. In attempting to explain this variation, the researchers used several data sources, including telephone interviews and a postal survey of participants [11]. The responses were used as explanatory variables in regression analyses, but they also could explain little of the variation in the main outcomes across practices. The authors concluded: "There is not a single explanation for the variation in change in practice or for the overall lack of change. A combination of organizational problems and lack of time and engagement is the most viable explanation for the lack of effect."

The COGENT-investigators evaluated a computerised clinical decision aid for guideline implementation, and conducted an interview study in parallel with their randomised trial [12]. The comments they received were predominantly negative, which served to explain the low level of use of the decision-aid system. Hetlevik and colleagues, in an earlier Norwegian trial, also observed a low use of their computerised clinical decision aid, and they failed to demonstrate an effect on blood pressure control [13]. In our study, the use of tools for cardiovascular risk assessment was low. However, the participants were generally positive when asked about their views of the decision aids we provided to them.

## Conclusion

Our multifaceted intervention targeting the professional behaviour of general practitioners was feasible to implement and was generally well received. However, while the intervention was effective in influencing prescribing, it did not impact on other outcomes. The data we collected do not provide compelling explanations for this. More use of qualitative research methods in the course of the trial could have increased our understanding of the trial results. Organisational factors are likely important to address in the development of interventions to improve the management of hypertension and hyperlipidaemia in primary care, and may have contributed to the lack of change that we observed for risk assessment and achievement of treatment goals. However, we do not have data to assess the extent to which this could help to explain our findings.

## Competing interests

The author(s) were the main investigators of the RaPP-trial.

## Authors' contributions

All authors participated in the planning of this study. AF and KH prepared the questionnaires with guidance from ADO. All authors participated in the interpretation of data. AF drafted the paper and conducted the statistical analyses.
